# Novel Synonymous Variant in *IL7R* Causes Preferential Expression of the Soluble Isoform

**DOI:** 10.1007/s10875-024-01688-8

**Published:** 2024-04-08

**Authors:** Rafah Mackeh, Yasmin El Bsat, Asha Elmi, Hani Bibawi, Mohammed Yousuf Karim, Amel Hassan, Bernice Lo

**Affiliations:** 1 Research Branch, Sidra Medicine, Doha, Qatar; 2 Division of Hematopathology, Sidra Medicine, Doha, Qatar; 3 College of Medicine, Qatar University, Doha, Qatar; 4 Pediatric Allergy and Immunology Department, Sidra Medicine, Ar-Rayyan, Qatar; 5 College of Health and Life Sciences, Hamad Bin Khalifa University, Doha, Qatar

**Keywords:** Primary immunodeficiency (PID), Severe Combined Immunodeficiency (SCID), IL-7Rα deficiency, IL-7Rα exon 6, Altered splicing, Synonymous variant

## Abstract

**Purpose:**

The interleukin-7 receptor (IL-7R) is primarily expressed on lymphoid cells and plays a crucial role in the development, proliferation, and survival of T cells. Autosomal recessive mutations that disrupt IL-7Rα chain expression give rise to a severe combined immunodeficiency (SCID), which is characterized by lymphopenia and a T^−^B^+^NK^+^ phenotype. The objective here was to diagnose two siblings displaying the T^−^B^+^NK^+^ SCID phenotype as initial clinical genetic testing did not detect any variants in known SCID genes.

**Methods:**

Whole genome sequencing (WGS) was utilized to identify potential variants causing the SCID phenotype. Splicing prediction tools were employed to assess the deleterious impact of the mutation. Polymerase Chain Reaction (PCR), Sanger sequencing, flow cytometry, and ELISA were then used to validate the pathogenicity of the detected mutation.

**Results:**

We discovered a novel homozygous synonymous mutation in the *IL7R* gene. Our functional studies indicate that this variant is pathogenic, causing exon 6, which encodes the transmembrane domain, to be preferentially spliced out.

**Conclusion:**

In this study, we identified a novel rare synonymous mutation causing a loss of IL-7Rα expression at the cellular membrane. This case demonstrates the value of reanalyzing genetic data based on the clinical phenotype and highlights the significance of functional studies in determining the pathogenicity of genetic variants.

**Supplementary Information:**

The online version contains supplementary material available at 10.1007/s10875-024-01688-8.

## Introduction

Primary immunodeficiencies (PIDs), also termed inborn errors of immunity (IEI), are inherited genetic anomalies leading to an immune dysregulation of the innate and/or adaptive immune system [1, 2]. In addition to being prone to infectious diseases, PID patients can present with autoimmunity, autoinflammation, allergies, and/or malignancies [3, 4]. Severe combined immunodeficiencies (SCIDs), classified as one of the most severe forms of PIDs, are life-threatening syndromes that present in infancy and are characterized by severe dysfunction of the adaptive immune system. SCIDs are usually fatal within the first two years of life if the patient’s immunity is not reconstituted in time. As a result, most SCID patients require a hematopoietic stem cell transplant (HSCT) [5, 6]. SCIDs can be classified based on the immunological phenotype into four groups including T^−^B^+^NK^+^, T^−^B^−^NK^+^, T^−^B^+^NK^−^, and T^−^B^−^NK^−^ [7].

IL-7R is predominantly expressed on lymphoid cells and plays a crucial role in the proliferation, differentiation, and survival of T cells [7, 8]. IL-7R is a heterodimer composed of the IL-7Rα chain (CD127) and the common gamma chain (γc/CD132) [8]. Upon binding of the ligand interleukin-7 (IL-7), the heterodimerization of these two chains activates downstream signaling pathways and upregulates the expression of cell cycle activation and anti-apoptotic genes [9, 10]. IL-7Rα is a 459 amino acid protein encoded by the *IL7R* gene located on the short arm of chromosome 5 (5p13.2) [11, 12]. It is comprised of the extracellular domain encoded by exons 1–5, the transmembrane domain encoded by exon 6, and the intracellular domain encoded by exons 7–8 [7]. IL-7Rα is expressed as two main isoforms: the transmembrane receptor (hereafter referred to as mIL-7Rα) encoded by the canonical full-length mRNA and the secreted soluble receptor (hereafter referred to as sIL-7Rα) encoded by an alternatively spliced mRNA lacking exon 6 [8, 12, 13].

Due to its critical role in T cell function, autosomal recessive mutations in IL-7Rα lead to a SCID phenotype typically characterized by severe T cell lymphopenia and normal to increased counts of B and NK cells, represented as T^−^B^+^NK^+^ SCID [11, 14, 15]. Most of the reported IL-7Rα mutations causing SCID are located within the extracellular domain with few reported in the intracellular domain [15].

In this study, we report two sisters exhibiting a T^−^B^+^NK^+^ SCID phenotype, significant maternofetal engraftment (MFE), and anemia. Whole genome sequencing (WGS) detected a novel homozygous synonymous mutation in exon 6 of the *IL7R* gene, which, although reported as likely benign, was predicted by *in silico* splice tools to enhance exon skipping. We investigated the impact of the mutation on the RNA and surface protein expression levels. Our data demonstrates that the synonymous variant is pathogenic, causing a preferential skipping of exon 6, leading to a deficiency in mIL-7Rα in the affected siblings.

## Methods

### Sample Collection

Informed consent was collected from all family members and healthy donor subjects who participated in this study according to the Institutional Review Board approved protocol (SIDRA Medicine IRB—protocol number 1601002512 approved on 30 June 2016). PBMCs were extracted using Ficoll-Paque PLUS (GE Healthcare) density gradient separation.

### Flow Cytometry

0.2 million PBMCs were resuspended in 1 × PBS (Thermofisher Scientific) and stained for 30 min on ice with the following monoclonal antibodies: PerCP-CD3 (BioLegend), PE-CD127 (BioLegend), V450 CD4 (clone RPA-T4, BD biosciences) and BV605-CD8 (clone SK1, BioLegend). Cells were acquired on NovoCyte flow cytometer and analyzed using FlowJo v10. MFI values were used for quantification and unpaired t-test was used for statistical analysis using Prism. * *p* < 0.05 ** *p* < 0.01 *** *p* < 0.001.

### RNA Extraction and RT-PCR

T cells in PBMCs were activated using beads coupled to anti-CD2, anti-CD3 and anti-CD28 beads (Miltenyi Biotec, Gaithersburg, MD, USA) and incubated for 3 days in 5% CO_2_ at 37 °C in advanced RPMI 1640 medium supplemented with 10% FBS and Penicillin/streptomycin. On day 3, cells were washed once and IL-2 was added to the media to allow the expansion of activated T cells. RNA was extracted from 2 × 10^6^ activated T cells on day 7. Briefly, cells were lysed using TRIzol lysis reagent (Invitrogen) and centrifuged for 15 min at 4 °C after phenol addition and mixing. Equal volume of 100% isopropanol was then added to the collected interphase containing RNA, and the RNA was precipitated at -20 °C overnight. The following day, precipitated RNA was spun down at 15000 g at 4 °C, washed once with 70% ethanol then eluted with water. Reverse transcription was then performed using the 5 × Iscript Reverse Transcription Supermix (Bio-Rad). IL-7Rα cDNA transcripts were amplified using HotStar Taq Master Mix (Qiagen) and the following primers: F-cDNA 5’ TCCAACCGGCAGCAATGTAT 3’ and R-cDNA 5’CTGGGCCATACGATAGGCTT 3’. PCR was performed on the 96-well thermal cycler (Thermofisher Scientific) at the following cycling conditions: 1 cycle of 95 °C for 10 min, 35 cycles of 95 °C for 30 s, 56 °C for 30 s, 72 °C for 30 s, and 1 cycle of 72 °C for 10 min. The amplified PCR products were loaded on 2% gel and visualized using CyberRed. The bands were purified using the QIAquick Gel extraction kit (Qiagen) for downstream Sanger Sequencing.

### Sanger sequencing

Genomic DNA (gDNA) was extracted either from granulocytes, EBV-transformed B cells, or peripheral blood using DNeasy® Blood & Tissue Kits (Qiagen, Germantown, MD, USA). PCR and Sanger sequencing were conducted using the following primers:

F-GGGTGAACATCCCTCTCATCA and R-ATGCCTTAATCCCCTTTGTGGT for genomic DNA, F-TCCAACCGGCAGCAATGTAT and R-CTGGGCCATACGATAGGCTT for cDNA. Sequences were analyzed using Unipro UGENE software using human reference genome GRCh37/hg19 as alignment reference. 

### ELISA

Human sCD127 levels were quantified in plasma samples or supernatants of PBMCs cultured for 24 h in complete medium using the Human IL-7 R alpha/CD127 ELISA Kit following the manufacturer’s instructions (Invitrogen, Cat# EH276RB). In summary, pre-coated 96-well plates were blocked for 1 h with 5% BSA in PBS then washed once with assay buffer. PBMCs supernatants or plasma samples were loaded and incubated overnight at 4 °C. sCD127 levels were detected using anti-human IL-7 R alpha Biotin Conjugate kept for 1 h, followed by 45 min incubation with streptavidin-HRP, and 30 min incubation with TMB substrate. The reaction was stopped with the stop solution and the plate was visualized with a plate reader at 450 nm. The standard curve was generated using the 4-parameter fit Microsoft Excel tool and the concentrations of the sCD127 were determined.

### Phosphoflow experiments (p-STAT5 detection)

0.5 million PBMCs were stimulated with 10 or 20 ng/mL of recombinant human IL7 (peprotech cat # 200–07). Cells were subsequently fixed with 1 × Lyse/Fix (BD Biosciences #558,049) and permeabilized with BD phosphoflow Perm Buffer III (BD biosciences #558,050) and stained with BV786 anti-human CD3 (BD biosciences #563,800), V450 anti-human CD4 (BD biosciences #561,838), APC-Cy7 anti-human CD8 (Biolegend# 344,714) and PE anti-pSTAT5 (BD biosciences #612,567). Stained cells were acquired on NovoCyte flow cytometer.

### *In Silico* splicing tools used

EX-SKIP (https://ex-skip.img.cas.cz) is a computational tool that estimates the probability of Exon skipping by comparing the ESE (exon splicing enhancer)/ESS (exon splicing silencer) ratio profile of a mutant sequence to the wild type [16]. Splice AI (https://spliceailookup.broadinstitute.org) is a non-proprietary machine learning structured tool that uses an algorithm to identify splicing variants. After inputting the targeted sequence, this tool gives a delta score for the acceptor and donor sites. Values generated are between 0 and 1.0, in which values close to 0 correspond to benign variants and values close to 1 are associated with pathogenic variants [17]. Human Splicing Finder (HSF) (https://www.genomnis.com/access-hsf) is a bioinformatics analysis software that utilizes 12 different algorithms to identify splicing motifs in the inserted sequence and predict the impact of variants on splicing signals. Splicing motifs would include the consensus branchpoint and auxiliary sequences such as ESS and ESE, in addition to, major splicing sites known as the donor and acceptor splice sites [18].

## Results

### Clinical history of the patients

The extended clinical summary of both patients is available in this article’s Online Resource 1. Briefly, P1, a female, was first noticed at 3 weeks of age when she presented to the pediatric ward with neck pustules and paronychia of two fingers in each hand. Her treatment included intravenous antibiotics, followed by a month of oral antibiotics. Initial immunological evaluations showed severely decreased CD4 + and CD8 + T cell counts, slightly decreased B cell count, and normal NK cell count. Re-evaluation at 6 weeks showed reduced CD4 + T cells, but normal CD8 + T cells, and elevated NK cell count, with similar findings at 4 months (summarized in Table 1). Her lymphocyte proliferation test showed severe reduction to PHA, ConA and Pok and a mild reduction to Candida. Suspected to have T^−^, B^+^, NK^+^ SCID, she was treated with antimicrobial prophylaxis including, PJP, fungal and viral prophylaxis in addition to immunoglobulin replacement therapy. At 4 months of age, she presented with oozing at the BCG site, low grade fever and mild cough. This resolved and then she remained reasonably well until age 12 months when she was admitted to the hospital with a history of persistent fever, recurrent skin facial rash and diarrhoea. Infection screen showed high CRP, chest X-ray showed pleural effusion, DFA was positive for entero/rhinovirus, corona NL63, and blood viral PCR was positive for CMV (Table 2). Unfortunately, despite optimum treatment with antibiotics, anti-fungal, anti-viral and anti-mycobacterial treatment, she continued to deteriorate with hypoxia that led to respiratory failure and death at the age of 18 months.

**Table 1:** Laboratory investigations in affected siblings

Parameter	P1		P2	
Age	3 weeks	16 weeks	3 weeks	12 weeks
Hemoglobin (g/L)	145 (125–205)	Not available	↑ 87 (125–205)	117 (90–140)
White Blood Count (10^9^/L)	6.3 (5–20)	Not available	8.7 (5–20)	6.4 (5–19.5)
Platelets (10^9^/L)	↑ 657 (150–400)	Not available	↑ 478 (150–400)	↑ 503 (150–400)
Neutrophils (10^9^/L)	2.8 (1–9.5)	Not available	4.9 (1–9.5)	1 (1–9)
Monocytes (10^9^/L)	↑ 2.4 (0.5–1.8)	Not available	↑ 3.2 (0.5–1.8)	1.1 (0.5–1.8)
Eosinophils (10^9^/L)	0.2 (0.2–0.6)	Not available	0.2 (0.2–0.6)	0.6 (0.2–0.6)
Basophils (10^9^/L)	0.1 (0.0–0.2)	Not available	0.1 (0.0–0.2)	0.1 (0.00–0.2)
Absolute Lymphocyte count	↓ 0.8 (2–17)	Not available	↓ 0.4 (2–17)	3.7 (2.5–16.5)
CD3 (%)	↓ 2.1 (60–85)	↓ 18.15 (51–77)	↓ 2.35 (53–84)	↓ 28.33 (53–84)
CD3 Absolute (cells/mcL)	↓ 29 (2300–7000)	↓ 872 (2500–5600)	↓ 14 (2500–5500)	↓ 874 (2500–5500)
CD3 + CD4 + (%)	↓ 0.9 (41–68)	↓ 3.4 (35–56)	↓ 1.84 (35–64)	↓ 8.39 (35–64)
CD3 + CD4 + Absolute (cells/mcL)	↓ 12 (1700–5300)	↓ 172 (1800–4000)	↓ 11 (1600–4000)	↓ 266 (1600–4000)
CD3 + CD8 + (%)	↓ 1.5 (9–23)	↑ 13.66 (12–23)	↓ 0.5 (12–28)	19.7 (12–28)
CD3 + CD8 + Absolute (cells/mcL)	↓ 21 (400–1700)	690 (590–1600)	↓ 3 (560–1700)	625 (560–1700)
CD19 + (%)	↓ 38.5 (4–26)	↑ 41.96 (11–41)	↑ 37.51 (6–32)	13.42 (6–32)
CD19 + Absolute	↓ 529 (600–1900)	1923 (430–3000)	↓ 227 (300–2000)	402 (300–2000)
CD3- CD56 + (%)	↓ 55.4 (3–23)	↑ 38.85 (3–14)	↓ 43.04 (4–18)	14.34 (4–18)
CD3- CD56 + Absolute (cells/mcL)	761 (200–1400)	↑ 1780 (170–830)	260 (170–1100)	431 (170–1100)
CD4/CD8 Ratio (%)	0.55	0.25	3.67	0.43
CD45RA (%)	↓ 9 (64–95)	Not available	Not available	↓ 0.3 (64–95)
CD45RO (%)	↑ 90.3 (2–22)	Not available	Not available	↑ 98.91 (2–22)
Immunoglobulin				
IgG (g/L) IgA (g/L) IgM (g/L)	↑ 10.05 (3.11–6.64) * ↑ < 0.50 (0.00–0.42) * ↑ 2.17 (0.00–1.27) *	↑ 15.17 (3.11–6.64) # 0.06 (0.00–0.42) # 0.89 (0.00–1.27) #	6.82 (1.62–8.72) * < 0.05 (0.0–0.1) * 0.07 (0.0–0.57) *	↑ 12.03 (1.1–6.5) # ↑ 0.79 (0.01–0.3) # 0.78 (0.3–0.9) #

Age-matched normal ranges shown in parentheses

^*^ Earliest result of Immunoglobulins data before IVIG administration (7 weeks old for P1; 3 days old for P2)

^#^ Immunoglobulins data after IVIG administration

Arrows indicate the variation compared to normal range

**Table 2:** History of infection in P1 and P2

Infection	P1	P2
Bacterial and/or viral infections encountered	**Bacterial infection:** Staphylococcus aureus **Viral infection:** Cytomegalovirus (CMV), Coronavirus NL63, Human Rhinovirus/Enterovirus, and BCG	**Bacterial infection:** Pseudomonas aeruginosa **Viral infection:** Cytomegalovirus (CMV), SARS-CoV-2, and Epstein-Barr Virus (EBV)

P1’s sibling, P2, born at term along with a twin brother, was not given BCG vaccine at birth as per the immunology recommendations. Her cellular testing at birth revealed very low CD4 + and CD8 + T cells with normal B and NK cells. However, subsequent testing at 3 weeks of age showed a significant increase in T cells, with normalization of CD8 + T cell numbers, suspected to be the result of MFE. This pattern was again seen at 4 months (Table 1). After referral for HSCT, a family donor search revealed that her healthy older brother was found to be a full HLA match. HSCT was done at 8 months of age, after which she demonstrated good immune reconstitution post-treatment (Table 3 and supplementary Fig. 1) and ceased immunoglobulin replacement therapy. Conditioning regimen included Treosulfan, Fludarabine and anti-thymocyte globulin (ATG). GVHD prophylaxis included cyclosporine and Mycophenolate mofetil (MMF). Post HSCT, she developed acute skin GVHD that was treated with a short course of steroids. P2 also had CMV reactivation 4 weeks post HSCT that was treated successfully with valganciclovir. She is currently growing well and following a post-HSCT vaccination schedule.

**Table 3:** Clinical data post-HSCT in P2

Parameter	3 Months Post HSCT	5 Months Post HSCT	9 Months Post HSCT	Normal Range
Age	11 Months	13 Months	17 Months	
Hemoglobin (g/L)	116	127	121	106–145
White Blood Count (10^9^/L)	↓ 4.3	7.5	10.4	6–16
Platelets (10^9^/L)	340	373	342	150–400
Neutrophils (10^9^/L)	1.2	2	2.8	0.6–5.1
Monocytes (10^9^/L)	0.9	0.7	0.6	0.2–1.4
Eosinophils (10^9^/L)	0.2	0.3	0.3	0.0–1.0
Basophils (10^9^/L)	0.0	0.1	0.1	0.0–0.2
Absolute Lymphocyte count (ALC)	↓ 1.2	2 ↓	6.6	2.7–12
CD3 (%)	68.75	63.59	72.31	53–75
CD3 Absolute (cells/mcL)	↓ 1307	2695	4264	2100–6200
CD3 + CD4 + (%)	↓ 14.42	↓ 18.34	42.84	32–51
CD3 + CD4 + Absolute (cells/mcL)	↓ 262	↓ 747	2513	1300–4300
CD3 + CD8 + (%)	↓ 46.25	↓ 41.13	25.17	14–30
CD3 + CD8 + Absolute (cells/mcL)	842	1676	1476	620–2000
CD19 + (%)	18.23	24.78	23.4	16–35
CD19 + Absolute	↓ 361	1091	1387	720–2600
CD3- CD56 + (%)	9.26	5.99	↓ 1.73	3–15
CD3- CD56 + Absolute (cells/mcL)	184	264	↓ 102	180–920
CD45RA/RO	0.31	0.45	1.7	
Immunoglobulin				
IgG (g/L) IgA (g/L) IgM (g/L)	Not Done Not Done Not Done	7.76 0.37 0.55	3.93 0.3 0.34	3–10 0.01–0.9 0.5–1.7
Infections	No infection detected	Cytomegalovirus (CMV)	No infection detected	

Arrows indicate the variation compared to normal range

The twin brother of P2 was found to have normal immunological markers, received standard vaccinations, and is in good health.

### Genetic findings

**Fig. 1:**
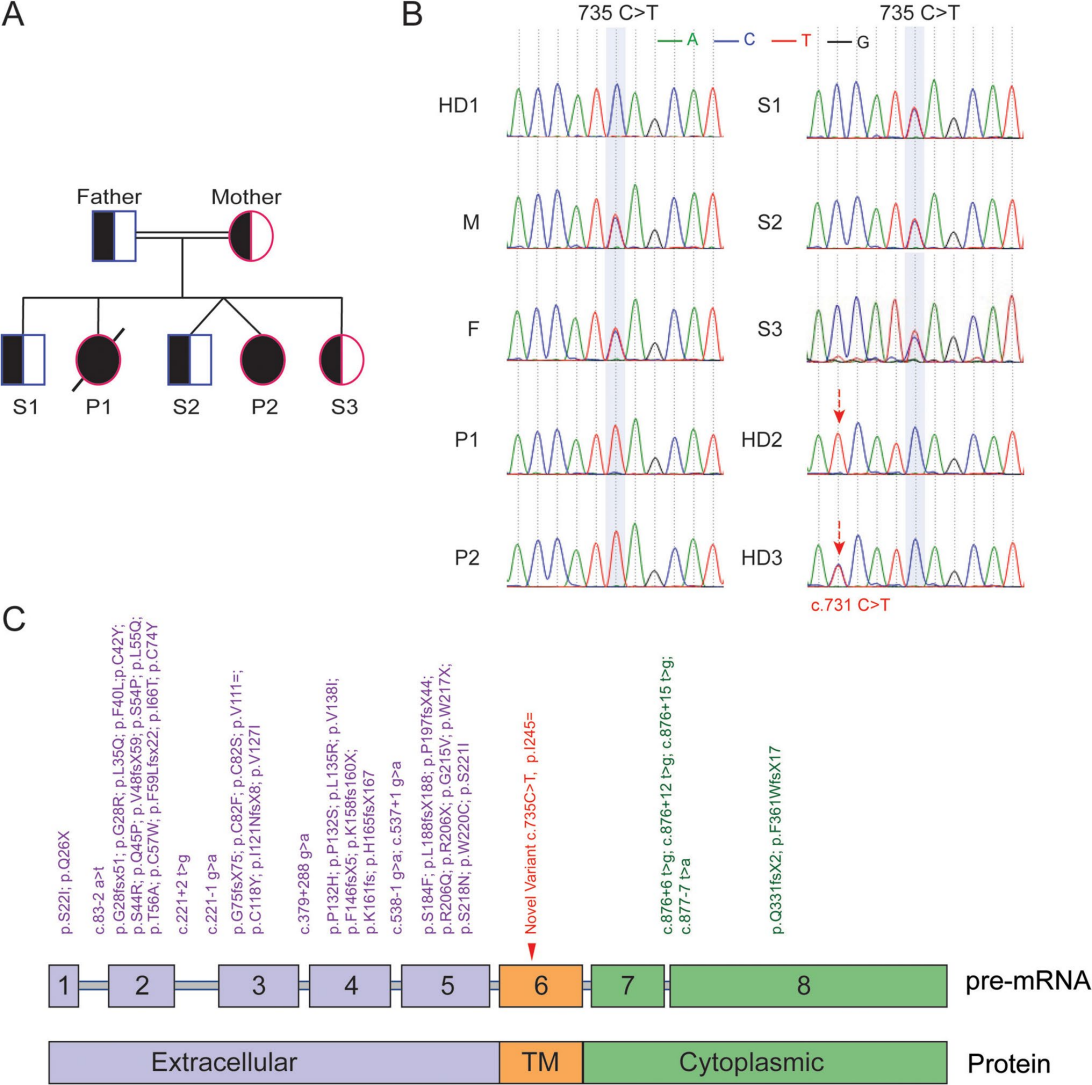
Patients are homozygous for a synonymous *IL7R* variant. **A**. Pedigree of the kindred. **B**. Sanger sequence confirmation of the c.735C > T mutation highlighted in light blue. Red arrows indicate a c.731C > T SNP in healthy donors. **C**. Diagram of IL-7Rα pre-mRNA and protein indicating the site of the c.735C > T mutation and listing the previously published SCID mutations associated with each exon/intron [11, 14, 15, 30, 33–53]

To understand the genetic basis of the patients’ disease, a blood sample from P1 was sent to Invitae for clinical PID gene panel testing but was unfortunately inconclusive due to contamination. A second sample was sent to Invitae and was also inconclusive for the same reason. Whole genome sequencing of DNA extracted from *in vitro* expanded T cell blasts from P1 failed to detect any homozygous rare variants despite parental consanguinity, indicative of a potential sample issue. These observations were later explained by the detection of 10% MFE in P1 blood as revealed by clinical testing at 14 months of age. Similarly, 16% MFE (81% of CD3^+^ T cells) was detected in P2 blood at 2 months of age. Therefore, to avoid maternal DNA contamination in the blood of P2, a saliva sample from P2 was sent to Invitae for PID gene panel testing. The Invitae results revealed a heterozygous pathogenic variant in *TRNT1* and heterozygous variants of uncertain significance in the genes *C9*, *FAT4* and *LYST*, none of which would explain the SCID phenotype in the patient. Therefore, WGS was performed on DNA extracted from whole blood of P2 (in which T cells accounted for only a small fraction of the cells at the time, thus the MFE was unlikely to interfere with the diagnosis) and a careful analysis in search of any rare variants in SCID genes was done. The analysis led to the detection of a novel homozygous synonymous mutation in exon 6 of the *IL7R* gene: c.735C > T (p.Ile245 =). Sanger sequencing performed using DNA extracted from whole blood showed that both P1 and P2 were homozygous for the mutation, while parents and healthy siblings were all heterozygous, consistent with an autosomal recessive mode of inheritance (Fig. 1A-C). This variant was not reported in any public database (e.g., ClinVar, Genome Aggregation Database (gnomAD), 1000 Genomes Project) at the time we started the functional investigations. However, the variant has recently been reported as likely benign by Invitae in ClinVar.

(https://www.ncbi.nlm.nih.gov/clinvar/variation/1896138/). Of note, the Sanger sequencing of two of the healthy donors (HDs) in Fig. 1B revealed a SNP c.731C > T (p.Thr244Ile), located three base pairs away from the patients’ mutation. HD2 was homozygous (T/T) while HD3 was heterozygous (C/T). This SNP had a minor allele frequency of > 20% in gnomAD and was classified as benign in ClinVar.

### c.735C > T variant causes a reduction in IL-7Rα membrane expression

**Fig. 2:**
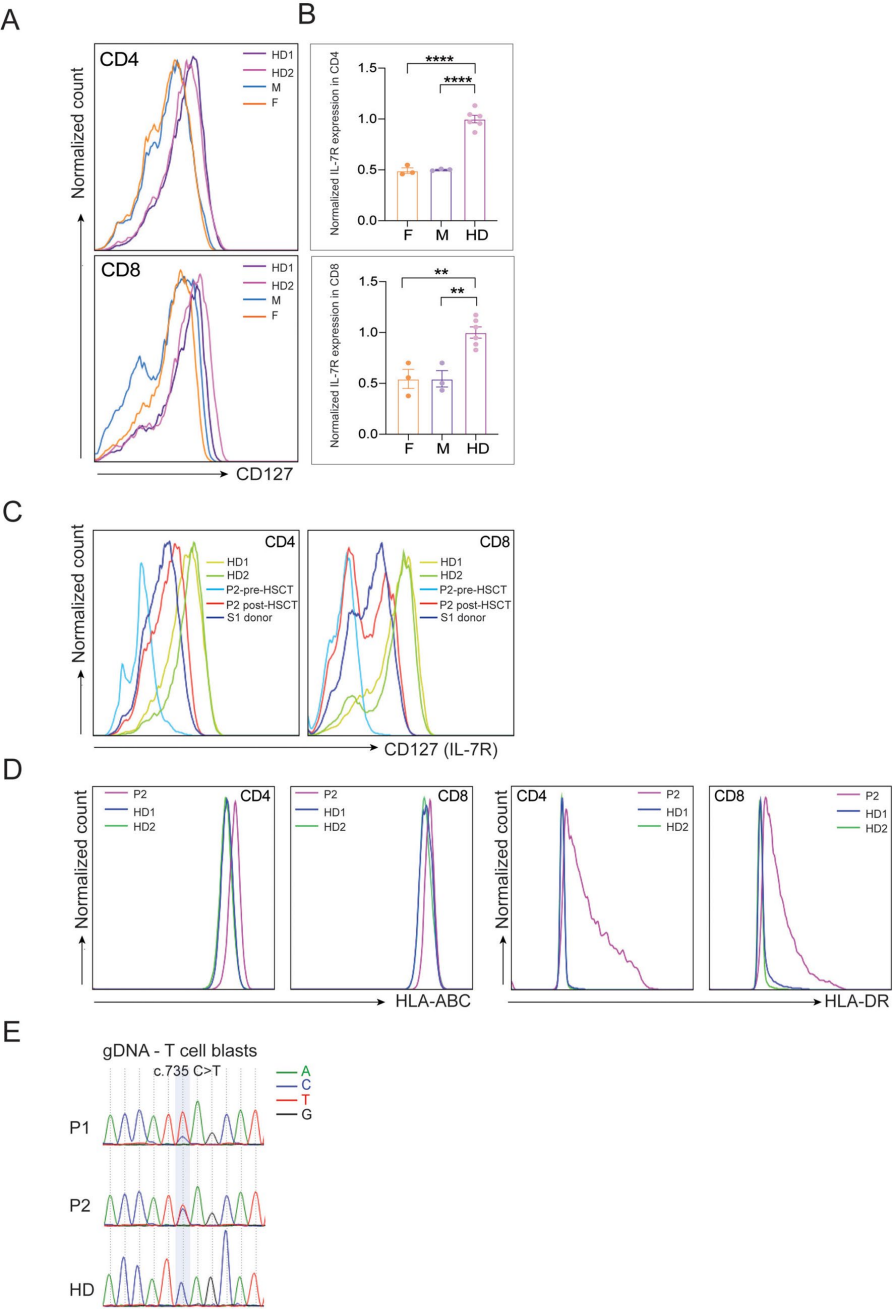
IL-7Rα expression is reduced by 50% in parents’ T cells. PBMCs from parents & 2 HDs were stained for IL-7Rα (CD127), CD3, CD4 and CD8. **A**. Histograms showing CD127 expression in CD3^+^ T cells. **B**. Quantification of IL-7Rα MFI in CD4 and CD8 from 3 independent experiments. IL-7R expression was normalized to the average of the HDs in each experiment. Unpaired T test was used for statistical analysis ** p < 0.01, **** p < 0.0001. **C**. Expression of CD127 pre- and post-HSCT on CD3^+^CD4^+^ or CD3^+^CD8^+^ PBMCs of P2 compared to the indicated individuals. **D**. Expression of HLA-A,B,C and HLA-DR in CD3^+^ CD4^+^ or CD3^+^CD8^+^ PBMCs from indicated individuals. **E**. Sanger sequencing of P1 and P2 gDNA extracted from *in vitro* expanded T cell blasts. Chromatogram shows the presence of the c.735C allele from the mother

To investigate the functional consequences of the novel mutation detected in our patients, we aimed to measure the expression of membrane IL-7Rα, also known as CD127, on the surface of the patients’ T cells. However, due to MFE, the majority of the T cells in the patients are of maternal origin. Therefore, we opted to measure CD127 on the parents’ T cells, which we hypothesized should have a 50% drop in expression compared to healthy individuals, consistent with the parents’ heterozygous genotype. As expected, CD127 expression was significantly reduced by 50% in both CD4 and CD8 T cell populations when compared to healthy donors (Fig. 2A&B), indicating that the mutation is altering IL-7Rα membrane expression. Interestingly, this reduction in the CD127 expression did not significantly affect downstream STAT5 phosphorylation upon different doses and time of IL-7 stimulation in the parents (Supplementary Fig. 2) possibly due to a compensatory mechanism.

As P2 was transplanted, we wanted to confirm the restoration of CD127 expression upon HSCT. As expected, percentage of CD3^+^ and CD4^+^ T cells, and recent thymic emigrants (CD45RA^+^CD31^+^) were restored to a similar level as her sibling donor (S1) (Supplementary Fig. 1). Similarly, expression of CD127 post-HSCT matched the level of the S1 donor. However, while CD127 expression prior to HSCT was expected to match the mother’s CD127 levels, we were surprised to see a severe reduction in the expression both in CD4 and CD8 T cells of P2 pre-HSCT (Fig. 2C). Because the expression of CD127 is high in resting T cells and is lost in activated or exhausted T cells [19–26], we hypothesized that maternal T cells that are engrafted in P2’s blood acquired an activated/exhausted state due to continuous alloantigen exposure. HLA class I/II staining was increased on CD4 and CD8 T cells in P2 compared to healthy donors, confirming the activated status of these cells, which explains the loss of CD127 on T cells in P2 (Fig. 2D). Finally, to rule out that the loss of CD127 observation was originating from the patient’s own T cells, we Sanger sequenced gDNA from the *in vitro* expanded T cell blasts from both P1 and P2. In line with the high degree of MFE, the sequences from both P1 and P2 T cells blast DNA showed the presence of the reference allele (C) at c.735 from the mother (Fig. 2E).

### c.735C > T causes a preferential splicing out of exon 6

IL-7Rα is expressed as a membrane-bound receptor (mIL-7Rα), encoded by the canonical transcript, and as a secreted protein (sIL-7Rα) encoded by an alternatively spliced transcript lacking exon 6. Given the location of the mutation within exon 6, and the nature of the variant (synonymous), we hypothesized that the mutation may interfere with splicing. We obtained predictions for the effect of the mutation on splicing using multiple tools. Raw results for both c.735 C > T and c.731 C > T mutations are shown in supplementary Fig. 3 and summarized in Table 4. With the Ex-Skip tool, the c.731 C > T SNP is predicted to increase the chances of Exon skipping with an ESS/ESE ratio of 1.06 versus 0.99 for the wild type sequence, while the Patients’ mutation c.735 C > T had an even higher probability of exon skipping with an ESS/ESE ratio of 1.18 (ESE is an exonic splicing enhancer sequence that enhances exon inclusion, while ESS is an exonic splicing silencer that inhibits exon inclusion. Thus, the higher the ESS/ESE ratio is, the more likely for an exon to be spliced out). The HSF tool showed that the c.735 C > T variant is in a splice acceptor site and the impact of the broken and created auxiliary sequences can be visualized on the graph and compared to c.731 C > T (Supplementary Fig. 3C). Finally, for the c.735C > T mutation, the Splice AI tool showed an increase in the delta score (0.21) for the acceptor loss prediction. Collectively, our *in silico* analysis predicted that the c.735C > T mutation may disrupt ESE elements and would create an ESS site, which would have an inhibitory effect on exon inclusion, and hence might lead to a higher chance of exon skipping than the WT allele. On the contrary, *in silico* analysis predictions for the c.731C > T SNP were inconsistent between tools (Table 4). A deeper search in the literature revealed that c.731C, also known as rs6897932, causes a twofold increase in the skipping of exon 6 and therefore twofold increase in the sIL‑7R versus the c.731 T allele [27].

**Fig. 3:**
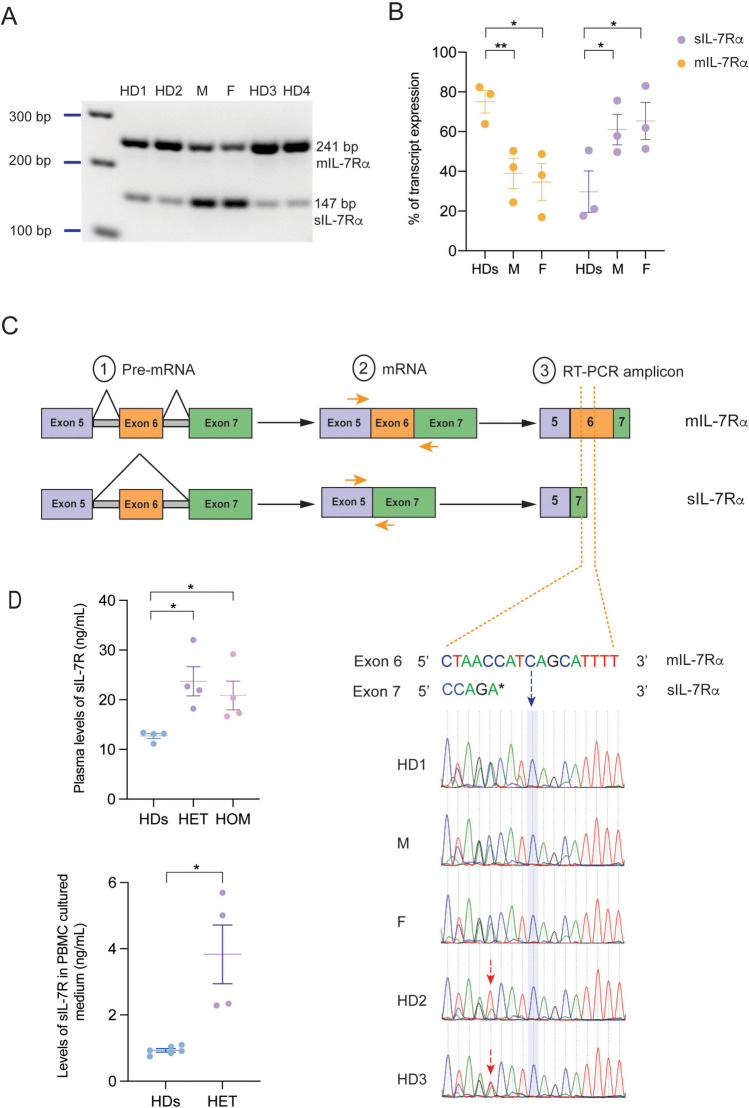
The c.735C > T mutation leads to exon 6 skipping. **A**. PCR products from both IL-7Rα transcripts. **B**. Transcript isoforms expression % from 3 independent experiments. **C**. Region flanked by orange arrows was Sanger sequenced. Chromatogram corresponds to the segment between the dashed lines where the end of exon 7 from the sIL-7Rα transcript overlaps with part of exon 6 from the mIL-7Rα. c.735C > T is highlighted in blue. Red dashed arrows indicate c.731C > T SNP observed in HD2 & HD3. * Indicates an inserted adenine by Taq-polymerase. **D**. Quantification of sIL-7Rα by ELISA in plasma (upper panel) of HDs, heterozygous parents (father, mother) or homozygous patients (P1 and P2), or in the medium of cultured PBMCs (lower panel) from HDs or heterozygous individuals (father and S1). Data shows two independent experiments for plasma, each done in duplicate and one replicate for culture supernatant done in technical duplicates. Unpaired T test was used for statistical analysis * p < 0.05, ** p < 0.01

 To validate the *in silico* predictions, we amplified the region between exon 5 and exon 7 (encompassing exon 6) by RT-PCR of T cell RNA from the parents and healthy donors to evaluate splicing of exon 6. Based on the set of primers used, the amplicon from. mIL-7Rα should be 241 bp and the amplicon from sIL-7Rα should be 147 bp (Fig. 3A). In line with the flow cytometry findings, the parents’ transcripts corresponding to the mIL-7Rα fragments were significantly reduced by 40–60% compared to the healthy donors, while the opposite pattern was observed for sIL-7Rα, consistent with the heterozygosity of the parents (Fig. 3B).

**Table 4:** *In silico* analysis of the consequence of the mutation on splicing using three different tools

Splice Tools	c.735 C > T, (I245=)			c.731 C > T, T244I		
**EX-SKIP**	**Seq-WT**	**Seq-Mut**		**Seq-WT**	**Seq-Mut**	
PESS (count)	3	3		3	3	
FASS-ESS hex2(count)	5	5		5	5	
FAS-ESS hex3 (count)	0	0		0	0	
IIE (count)	38	38		38	38	
IIE (sum)	781.0494	781.0494		781.0494	781.0494	
NI-ESS trusted (count)	21	21		21	21	
NI-ESS all (sum)	–27.5791	–28.0005		–27.5791	–27.6290	
PESE (count)	4	0		4	1	
RESCUE-ESE (count)	12	11		12	13	
EIE (count)	28	27		28	26	
EIE (sum)	402.6133	396.7954		402.6133	389.0706	
NI-ESE trusted (count)	24	19		24	23	
NI-ESE all (sum)	32.4083	27.5524		32.4083	31.3976	
ESS (total)	67	67		67	67	
ESE (total)	68	57		68	63	
ESS/ESE (ratio)	0.99	1.18		0.99	1.06	
**SpliceAI**	**Score**			**Score**		
Acceptor Loss	0.21			0.00		
Donor Loss	0.00			0.00		
Acceptor Gain	0.00			0.02		
Donor Gain	0.00			0.00		
**Human Splicing Finder (HSF)**	**Position**	**Sequence**	**Status**	**Position**	**Sequence**	**Status**
EIE	Chr5:35874472	ACCATC	Broken	Chr5:35874469	CTAACC	Broken
				Chr5:35874472	ACCATC	Broken
PESE	Chr5:35874472	ACCATCAG	Broken	Chr5:35874472	ACCATCAG	Broken
	Chr5:35874476	TCAGCATT	Broken			
RESCUE ESE	Chr5:35874474	CATCAG	Broken	Chr5:35874473	TCATCA	Created
ESE_SRp40	Chr5:35874473	CCATTAG	Created	N/A	N/A	N/A
ESS_hnRNPA1	Chr5:35874477	TAGCAT	Created	N/A	N/A	N/A
Predicted Impact	Alteration of Auxiliary Sequences: Significant alteration of ESE/ESS motifs ratio (-4)			Alteration of Auxiliary Sequences: Significant alteration of ESE/ESS motifs ratio (-2)		

The *in silico* splicing predictions along with the experimental results suggest that the c.735C > T mutation causes preferential splicing out of exon 6 from the affected allele. To confirm, we Sanger sequenced the cDNA from the parents and the HDs, including HD2 and HD3 with the c.731C > T SNP. If the c.735C > T mutation causes constitutive skipping of exon 6, then the mIL-7Rα transcript should be solely or predominantly derived from the WT allele, and thus the mutation would be absent from the cDNA as the exon bearing the mutation would be spliced out. As expected, Sanger sequencing of the *mIL7Ra* cDNA revealed a homozygous WT sequence for the parents in which the c.735C > T mutation was not detected, while the c.731C > T SNP was present in the cDNA sequence of HD2 and HD3 as indicated by the red arrows in the figure, which was consistent with their genotype (Fig. 3C). To further confirm that the increase in the sIL-7Ra transcript leads to an increase at the protein level, we measured by ELISA, the concentration of sIL-7Rα secreted in the plasma of P1, P2, father, mother, and HDs as well as in the medium of cultured PBMCs from the heterozygous father and sibling. Our results show a clear increase in the sIL-7Ra in heterozygous individuals compared to HD in both plasma and supernatants. Interestingly, homozygous P1 and P2 showed similar levels of sIL-7Rα in plasma as parents despite the severe lymphopenia. In a previous study, monocytes (CD14 + cells) were shown to secrete sIL-7Rα upon LPS or TNF stimulation [28]. Therefore, one explanation for the high secretion of sIL-7Rα observed in P1 and P2 despite the lymphopenia, maybe the potential high secretion by the monocytes, given that both patients had encountered bacterial infections.

Collectively, our data show that the c.735C > T mutation causes a preferential skipping of exon 6, which leads to the exclusive expression of the soluble form of IL-7Rα and the loss of the functional membrane bound form. Therefore, both P1 and P2 who are homozygous for the mutation, have IL-7Rα deficiency due to the lack of mIL-7Rα.

## Discussion

In this study, we delved into a perplexing case involving two sisters with T^−^B^+^NK^+^ SCID. Through careful analysis of the whole genome sequencing data, we uncovered a rare and novel synonymous variant in the *IL7R* gene. Despite the variant being synonymous and considered to be likely benign in ClinVar, we harbored suspicion regarding its pathogenicity since the patients’ phenotype and the mode of inheritance were consistent with IL-7Ra deficiency.

Synonymous mutations have increasingly been identified as disease-causative variants in numerous diseases [29]. In the case of SCID, only a few synonymous mutations have been reported as pathogenic, with only one occurring in IL-7Rα [30]. The reported c.333 T > A, p.V111V variant was found to create an active cryptic splice site within exon 3, resulting in deletion of 49 nucleotides. This deletion caused a frameshift and introduced an early stop codon at residue 119. Similarly, the c.735C > T variant identified in this study also disrupts the conventional splicing of IL-7Ra leading to preferential skipping of exon 6. A similar mechanism has been reported in a study linking the c.731C *IL7R* polymorphism to susceptibility to multiple sclerosis (MS) [27]. The authors showed that the C risk allele (rs6897932) results in a two-fold increase in the exclusion of exon 6 compared to the T allele, presumably due to an augmented ESS. Interestingly, we did not observe an increase in soluble IL-7Rα isoform abundance in the healthy donors homozygous for the C allele versus those carrying the c.731C > T SNP. Studies investigating the rs6897932 SNP have shown that this risk allele appears to be associated with MS only in the European population, but not in Turkish nor Middle Eastern populations [31, 32]. It is plausible that another factor, e.g. another SNP, in these populations may influence this exon 6 skipping. Conducting additional association studies in various ethnicities might provide a more comprehensive understanding of the functional consequences of this SNP.

It is important to note that the high degree of MFE impeded clinical testing, resulting in a delay in diagnosis and obstructed the execution of functional assays using the patients' T cells. Therefore, for SCID cases with suspected or confirmed MFE, it may be prudent to consider a saliva sample for clinical genetic testing, in order to avoid maternal DNA contamination.

Our report illustrates of how the synergistic employment of next-generation sequencing (NGS), computational tools, and functional assays empowers the identification of pathogenic variants. This study further highlights the importance of reanalyzing genomic data guided by the clinical phenotype as well as the crucial role of functional studies in validating the pathogenicity of variants. Since this variant had been classified in ClinVar as likely benign, it was even more critical to provide functional data demonstrating that this variant is not benign but in fact pathogenic and causative of a life-threatening disease.

## Conclusions

We identified a novel rare synonymous mutation in IL-7Rα causing a preferential expression of the soluble isoform of IL-7Rα. Our work offers a roadmap/pipeline for similar complex SCID cases with maternal engraftment and highlights the importance of re-analyzing genomics data guided by the phenotype when initial testing is negative.

### Supplementary Information

Below is the link to the electronic supplementary material.

Supplementary file1 (DOCX 16 KB)

Supplementary file2 Supplementary Figure 1. HSCT restores RTEs in P2. PBMCs were stained for the indicated surface markers to assess the restoration of recent thymic emigrants. (TIF 36099 KB)

Supplementary file3 Supplementary Figure 2. p-STAT5 is not affected in heterozygous parents. (A) PBMCs from HDs, father or mother were stimulated for 20 minutes with 20 ng/mL of IL-7 and subsequently stained for CD3, CD4, CD8 and p-STAT5. Bar histograms show the mean fluorescence intensity of p-STAT5 in CD3+ CD4+ or CD3+ CD8+ T cells. (B) PBMCs from HDs or father were stimulated for the indicated times with 20 ng/mL of IL-7 and subsequently stained for CD3, CD4, CD8 and p-STAT5. (C) PBMCs from HDs or father were stimulated for 10 minutes with 10 ng/mL of IL-7 and subsequently stained for CD3, CD4, CD8 and p-STAT5. Bar histograms show the mean fluorescence intensity of p-STAT5 in CD3+ CD4+ or CD3+ CD8+ T cells. (TIF 35723 KB)

Supplementary file4 Supplementary Figure 3. In silico predictions using splicing prediction tools. (A) Ex-SKIP tool showing the ESS/ESE ratio for the c.731 C>T and c.735 C>T variants in comparison to WT. Red box indicated the location of the variant. (B) Graphical representation from the Human Splicing Finder tool for both mutations showing that c.735 C>T is in a splice acceptor site. Impact on Auxiliary sequences (e.g. ESEs) is also shown. (C) Splice AI tool showing a higher Δ score for the acceptor loss of 0.21 for c.735 C>T compared to 0 for c.731 C>T. (TIF 36835 KB)

## Data Availability

Data sharing not applicable to this article as no datasets were generated or analysed during the current study.
