# Supplementary material for: Novel Synonymous Variant in IL7R Causes Preferential Expression of the Soluble Isoform
Source: J Clin Immunol. 2024 Apr 8;44(4):96. doi: 10.1007/s10875-024-01688-8 (PMC11001715; doi:10.1007/s10875-024-01688-8)
Supplement: Supplementary file 1 — Supplementary file1 (DOCX 16 KB) [file 10875_2024_1688_MOESM1_ESM.docx]

Novel Synonymous Variant in *IL7R* Causes Preferential Expression of the Soluble Isoform

Rafah Mackeh^1*^, Yasmin El Bsat^1*^, Asha Elmi^1^, Hani Bibawi^3^, Mohammed Yousuf Karim^3,4^, MBBChir, Amel Hassan^2^, and Bernice Lo^1,5#^

1: Research Branch, Sidra Medicine, Doha, Qatar

2: Pediatric Allergy and Immunology Department, Sidra Medicine, Qatar

3: Division of Hematopathology, Sidra Medicine, Doha

4: College of Medicine, Qatar University

5: College of Health and Life Sciences, Hamad Bin Khalifa University, Doha, Qatar.

* Equal contribution

# Correspondence:

Dr. Bernice Lo

Sidra Medicine

Education City, North Campus

Doha, Qatar

Email: blo@sidra.org

Tel: +974-4003-7385

**Online Resource 1**

**Extended clinical summary of the patients**

Patient P1, a female, was first noticed at 3 weeks of age when she presented to the pediatric ward with neck pustules and paronychia of two fingers in each hand. Her treatment included intravenous antibiotics, followed by a month of oral antibiotics.

Initial immunological evaluation showed severely decreased CD4+ and CD8+ T cell counts, slightly decreased B cell count, and normal NK cell count. Re-evaluation at 6 weeks showed reduced CD4+ T-cells, but normal CD8+ T-cells, and elevated NK cell count, with similar findings at 4 months (summarized in **Table 1**).

Her lymphocyte proliferation showed severe reduction to PHA, ConA and Pok and mild reduction to Candida. Thus, P1 was suspected to have a T^-^, B^+^, NK^+^ SCID and was treated with antimicrobial prophylaxis including, PJP, Fungal and viral prophylaxis in addition to Immunoglobulin replacement therapy. Her blood was sent twice for genetic testing but both attempts were inconclusive due to DNA contamination. Attempted saliva collection has failed to get the optimum amount required for DNA testing. Testing for MFE in whole blood of P1 revealed 8.5% MFE. Of note, her absolute lymphocyte count at birth and until 3 weeks of age was low at 0.8, repeated complete blood count at age 6 weeks showed an increase to 9.0 and continued to be normal to high. She then grew well until 4 months of age when she then presented with oozing at the BCG site, low grade fever and mild cough. This resolved and she then remained reasonably well until age 12 months when she was admitted to the hospital with a history of persistent fever, recurrent skin facial rash and diarrhoea. Infection screen showed high CRP, chest X-ray showed pleural effusion, DFA was positive for entero/Rhinovirus and corona NL63, and blood viral PCR was positive for CMV. Abdominal ultrasound revealed enlarged liver with focal lesions and marginally enlarged spleen. Liver enzymes and albumin were normal. MRI confirmed the enlarged liver with focal lesions, small right pleural effusion, a few retropharyngeal non-significant lymph nodes noted. Fever persisted, but blood cultures remained negative. She was suspected to have disseminated BCGiosis, however 3 gastric aspirations were performed, with all 3 mycobacterial PCR and smear being negative. Because of the persistent fever and rising CRP despite optimum antibacterial and antifungal treatment she was given 4 anti-mycobacterial medications orally along with intravenous amikacin. She also commenced systemic treatment for CMV viraemia, CMV viral load being between 250 to 5444 copies/mL. She developed CMV retinitis which was treated by the retinitis specialist ophthalmology consultant with intra-vitreous ganciclovir. Unfortunately, despite optimum treatment with antibiotics, anti-fungal, anti-viral and anti-mycobacterial treatment, she continued to deteriorate with hypoxia that led to respiratory failure and death at the age of 18 months.

P1’s sibling, P2, born along with a twin brother, at term, was not given any BCG at birth as per the immunology recommendations. Her cellular testing at birth revealed very low CD4+ and CD8+ T cells with normal B and NK cells. However, subsequent testing at 3 weeks of age showed a significant increase in T cells, with normalization of CD8+ T-cell numbers, suspected to be the result of MFE. This pattern was again seen at 4 months (**Table 1**). Subsequent investigation confirmed the presence of 16% MFE in whole blood. After referral for HSCT, a family donor search revealed that her healthy older brother was found to be a full HLA match. After HSCT, she demonstrated good immune reconstitution post-treatment and ceased immunoglobulin replacement therapy. She is currently well and following a post-HSCT vaccination schedule. The twin brother of P2 was found to have normal immunological markers, received standard vaccinations, and is in good health.
