# Supplementary figures and images for: Novel Synonymous Variant in IL7R Causes Preferential Expression of the Soluble Isoform
Source: J Clin Immunol. 2024 Apr 8;44(4):96. doi: 10.1007/s10875-024-01688-8 (PMC11001715; doi:10.1007/s10875-024-01688-8)

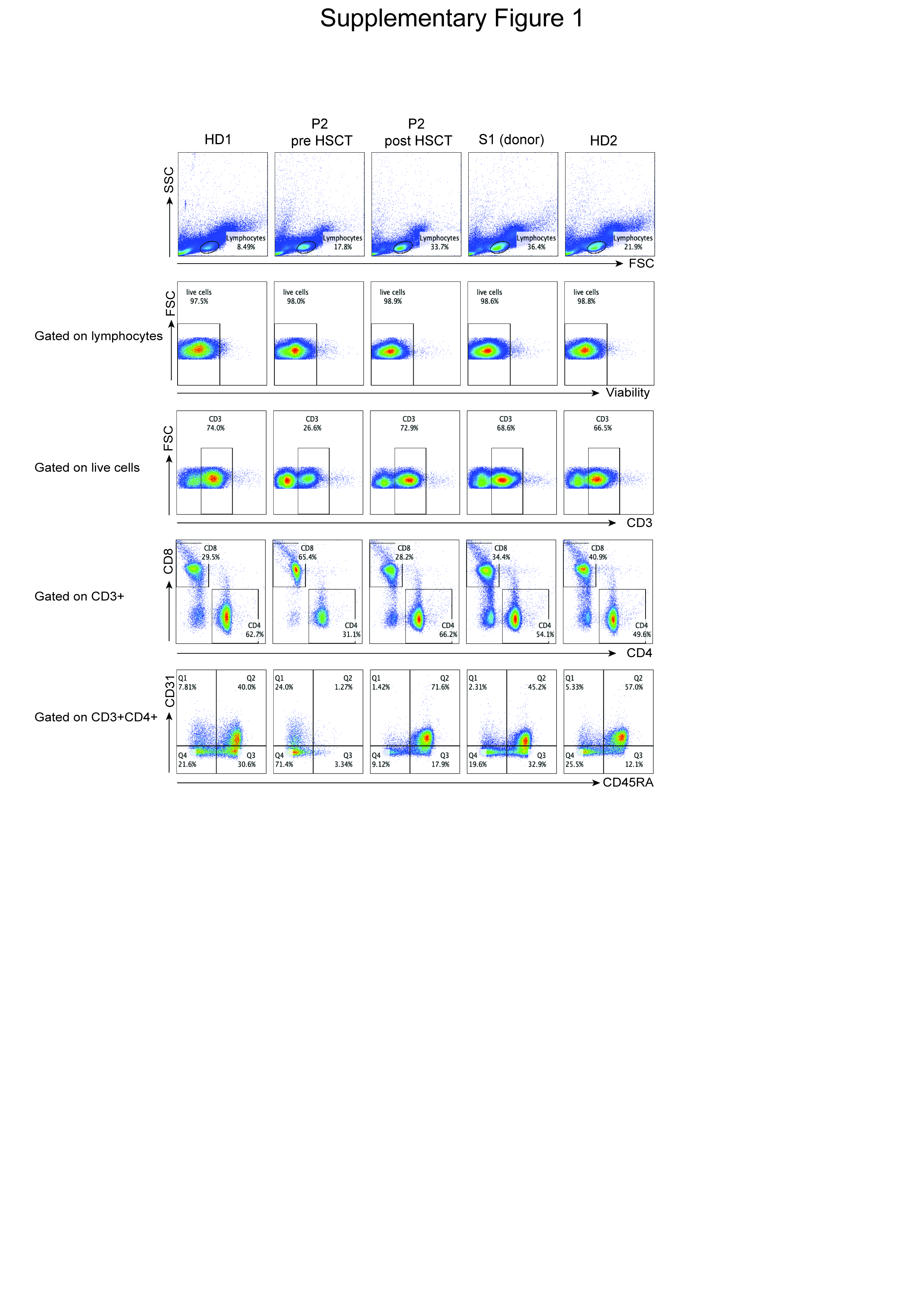

Supplement: Supplementary file 2 — Supplementary file2 Supplementary Figure 1. HSCT restores RTEs in P2. PBMCs were stained for the indicated surface markers to assess the restoration of recent thymic emigrants. (TIF 36099 KB) [file 10875_2024_1688_MOESM2_ESM.tif]

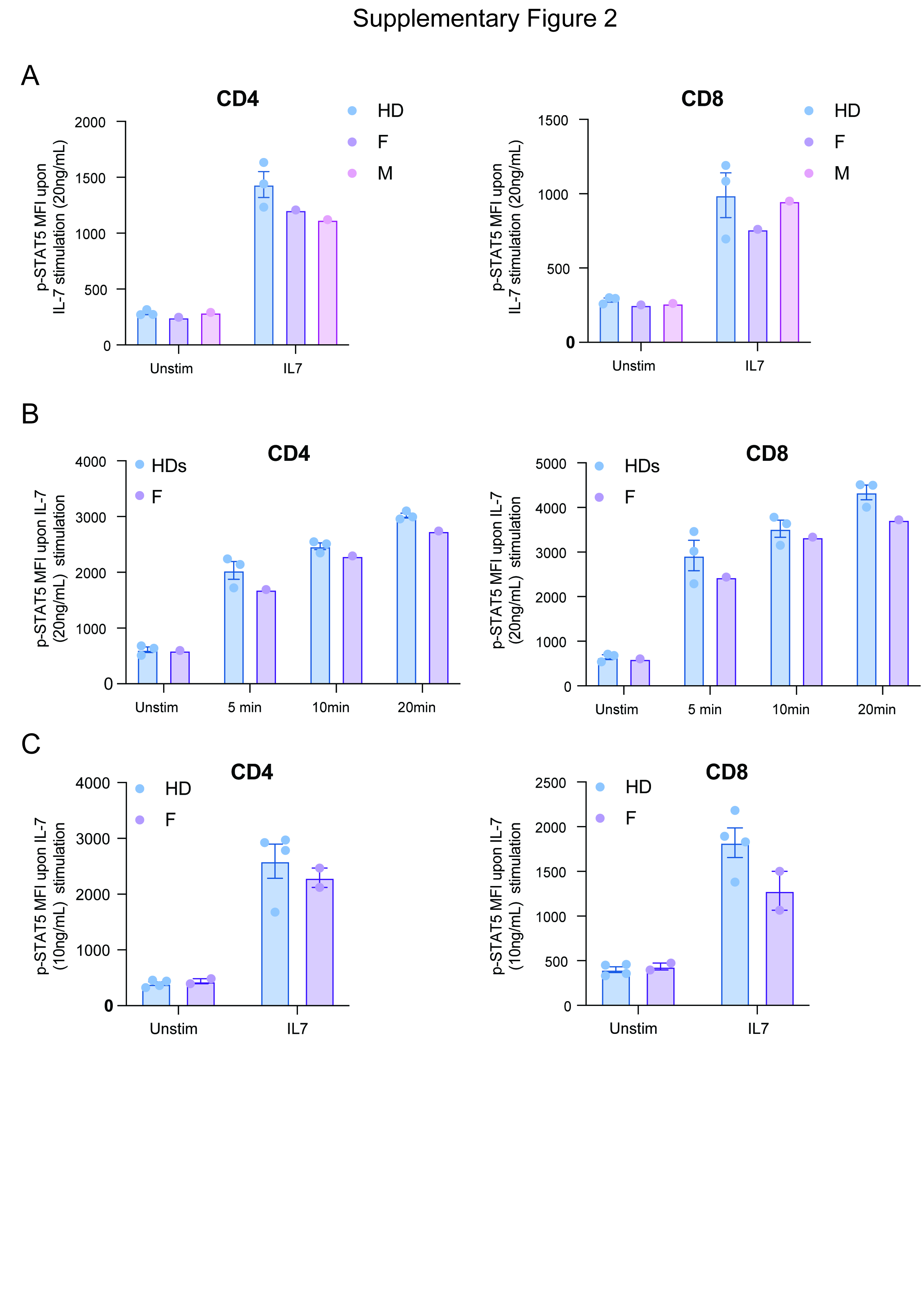

Supplement: Supplementary file 3 — Supplementary file3 Supplementary Figure 2. p-STAT5 is not affected in heterozygous parents. (A) PBMCs from HDs, father or mother were stimulated for 20 minutes with 20 ng/mL of IL-7 and subsequently stained for CD3, CD4, CD8 and p-STAT5. Bar histograms show the mean fluorescence intensity of p-STAT5 in CD3+ CD4+ or CD3+ CD8+ T cells. (B) PBMCs from HDs or father were stimulated for the indicated times with 20 ng/mL of IL-7 and subsequently stained for CD3, CD4, CD8 and p-STAT5. (C) PBMCs from HDs or father were stimulated for 10 minutes with 10 ng/mL of IL-7 and subsequently stained for CD3, CD4, CD8 and p-STAT5. Bar histograms show the mean fluorescence intensity of p-STAT5 in CD3+ CD4+ or CD3+ CD8+ T cells. (TIF 35723 KB) [file 10875_2024_1688_MOESM3_ESM.tif]

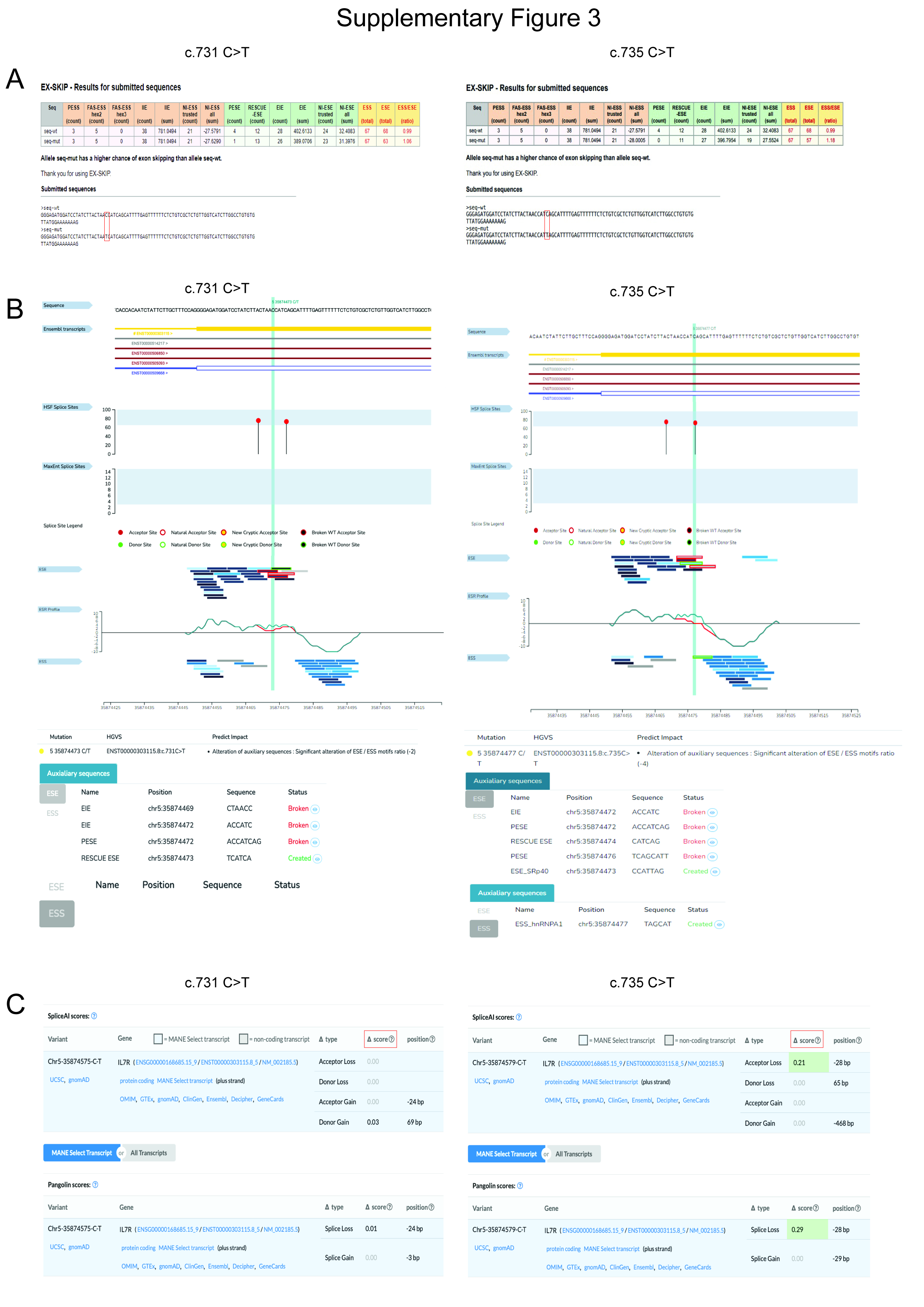

Supplement: Supplementary file 4 — Supplementary file4 Supplementary Figure 3. In silico predictions using splicing prediction tools. (A) Ex-SKIP tool showing the ESS/ESE ratio for the c.731 C>T and c.735 C>T variants in comparison to WT. Red box indicated the location of the variant. (B) Graphical representation from the Human Splicing Finder tool for both mutations showing that c.735 C>T is in a splice acceptor site. Impact on Auxiliary sequences (e.g. ESEs) is also shown. (C) Splice AI tool showing a higher Δ score for the acceptor loss of 0.21 for c.735 C>T compared to 0 for c.731 C>T. (TIF 36835 KB) [file 10875_2024_1688_MOESM4_ESM.tif]
